# Role of Non-Myocyte Gap Junctions and Connexin Hemichannels in Cardiovascular Health and Disease: Novel Therapeutic Targets?

**DOI:** 10.3390/ijms19030866

**Published:** 2018-03-15

**Authors:** Robert D. Johnson, Patrizia Camelliti

**Affiliations:** School of Biosciences and Medicine, University of Surrey, Guildford GU2 7XH, UK

**Keywords:** connexin, hemichannel, gap junction, cardiovascular disease, fibroblast, endothelial, macrophage, non-myocyte, therapeutic, inflammation

## Abstract

The heart is a complex organ composed of multiple cell types, including cardiomyocytes and different non-myocyte populations, all working closely together to determine the hearts properties and maintain normal cardiac function. Connexins are abundantly expressed proteins that form plasma membrane hemichannels and gap junctions between cells. Gap junctions are intracellular channels that allow for communication between cells, and in the heart they play a crucial role in cardiac conduction by coupling adjacent cardiomyocytes. Connexins are expressed in both cardiomyocytes and non-myocytes, including cardiac fibroblasts, endothelial cells, and macrophages. Non-myocytes are the largest population of cells in the heart, and therefore it is important to consider what roles connexins, hemichannels, and gap junctions play in these cell types. The aim of this review is to provide insight into connexin-based signalling in non-myocytes during health and disease, and highlight how targeting these proteins could lead to the development of novel therapies. We conclude that connexins in non-myocytes contribute to arrhythmias and adverse ventricular remodelling following myocardial infarction, and are associated with the initiation and development of atherosclerosis. Therefore, therapeutic interventions targeting these connexins represent an exciting new research avenue with great potential.

## 1. Introduction

The heart is a complex multicellular organ composed of cardiomyocytes (CMs) and non-myocytes, including cardiac fibroblasts (CFs), endothelial cells (ECs), smooth muscle cells (SMCs), pericytes, resident stem cells and immune cells. Each cell population has distinct features and functions, and they work closely together to determine the structural, biochemical, mechanical and electrophysiological properties essential for maintaining effective myocardial function [1]. CMs are muscle cells responsible for generating contractile force [2]; CFs produce and remodel the extracellular matrix (ECM) in response to physiological or pathological stimuli [3]; whilst ECs form the cardiac endothelium, the interior lining of blood vessels and cardiac valves [1]. Immune cells, such as monocytes and macrophages, are also found in the heart, where they are recruited from the blood following cardiac injury to aid wound-healing [4], but recent evidence has shown that populations of cardiac tissue-resident macrophages also exist, and that they are involved in tissue homeostasis [5,6]. Interestingly, CMs occupy 70–85% of the myocardial tissue volume [7], but only constitute around 30% of the actual cell numbers in the heart [8,9], with non-myocytes comprising the remaining 70% of cells [9]. CFs are generally believed to be the largest population of non-myocytes [10], contributing up to two thirds of total cells in rat [11] and human [12] hearts. However, a recent study has argued that ECs are the dominant non-myocyte population, accounting for 60% of total non-myocytes, whilst CFs constitute under 20% of non-myocyte cells [13]. This has led to debate over the cellular composition of the non-myocyte population in the heart, and the relevance of each cell type in cardiac homeostasis and cardiovascular disease.

In the adult heart, CMs are arranged in highly organised muscle sheets and are interconnected by intercalated discs, specialised cell junctions responsible for maintaining cardiac tissue structure integrity and allowing synchronised contraction [14]. Intercalated discs contain three distinct components; desmosomes, fascia adherens, and gap junctions. Desmosomes and fascia adherens are mechanical linkages, anchoring cell membranes to the intermediate filament network and the actin cytoskeleton respectively, whilst gap junctions form dynamic intracellular communication channels [14]. Gap junctions mediate cell-to-cell movement of ions and are crucial for impulse conduction through the cardiac conduction system and ventricular myocardium [15]. Gap junctions are also involved in the transfer of metabolites/second messengers between cells and allow sharing of metabolic demands across groups of cells [16]. Gap junctions are formed when a hemichannel in the plasma membrane of one cell docks with a hemichannel in the plasma membrane of an adjacent cell [17], with hemichannels made up of six connexin protein subunits [18]. A variety of connexins are expressed in the cardiovascular system, including connexin (Cx)31.9, Cx32, Cx37, Cx40, Cx43, and Cx45, although Cx37, Cx40, Cx43, and Cx45 are the predominant connexin isoforms [19]. Connexin expression also shows regional differences both in the heart and in the vasculature. For example, in the heart, Cx43 is found mainly between atrial and ventricular myocytes, as well as in parts of the conduction system, Cx40 is expressed in atrial myocytes, the atrioventricular node, bundle of His and ventricular conduction system, and Cx45 is mainly expressed in the sinoatrial node (SAN), atrioventricular node (AVN), bundle of His and bundle branches [15].

Normal heart rhythm is dependent on the coupling of CMs by gap junctions, with connexin and gap junction remodelling leading to potentially serious, and often fatal, cardiac arrhythmias [20]. The possible roles of Cx43 and Cx40 remodelling have been well-studied, but Cx45 remodelling is less understood [21]. Down-regulation of Cx43 has been observed in the failing human heart [22,23], whilst knockout of Cx43 in mice increases the incidence of sudden cardiac death resulting from spontaneous ventricular arrhythmia [24], and increases the frequency and length of ventricular tachycardia induced by ischemia [25]. Further, dephosphorylation and translocation of Cx43 from gap junctions to the cell cytosol has been shown to contribute to electrical uncoupling after ischemia in a Langendorff-perfused rat heart [26]. Mutations in Cx40 have been linked to patients with atrial fibrillation [27,28,29], whilst transgenic mice harbouring a human loss-of-function Cx40 mutation display reduced atrial conduction and prolonged incidence of atrial fibrillation as a result of reduced gap junctional conductance [30]. However, investigations in to the possible role of Cx45 in cardiac disease have been hampered by Cx45 knockout in animals causing an endocardial cushion defect in early cardiogenesis, and death shortly after birth [31]. A mouse model with Cx45 deletion specifically in adult CMs has been developed and has shown that Cx45 is important in AVN conduction, but is not essential for the survival of adult mice [32].

Dysfunctional hemichannels have also been associated with cardiac disease. Specifically, two atrial fibrillation-linked Cx40 mutations, V85I and L221I, were shown to cause increased hemichannel conductance without changing gap junction function [33]. This gain-of-function mutation is thought to contribute to atrial fibrillation by increasing the influx and outflux of sodium (Na^+^) and potassium (K^+^) ions respectively, leading to membrane depolarisation, Na^+^ channel inactivation, and reduced CM excitability [33]. In addition, the G38D Cx40 mutation, another atrial fibrillation-associated mutation, is also thought to increase hemichannel function [34]. Cx43 hemichannels in CMs have been implicated in exacerbating ischemia-reperfusion injury, with Cx43 hemichannel blockade by the peptide Gap19 improving CM viability both in vitro and in vivo [35].

Therefore, connexins are crucial for the maintenance of normal cardiac function. However, as CMs are not the only cell type to express connexins in the heart and not the dominant cardiac cell population in terms of cell numbers, it is important to consider what roles non-myocyte connexins play in cardiovascular function. This review highlights the role of CF, EC, and macrophage connexins in the healthy and diseased heart, and discusses how modifying their function may lead to new therapeutic benefits.

## 2. Connexins and Cardiac Fibroblasts

### 2.1. Fibroblast Identification and Function

CFs are widely distributed cells of mesenchymal origin that are traditionally defined as cells that secrete ECM components, such as different types of collagen and fibronectin [36]. However, collagen production is not used as a defining characteristic of CFs, with morphological identifiers used instead. Morphologically, CFs are flat, spindle-shaped cells that lack a basement membrane, but display irregular folding and multiple elongated cytoplasmic processes/sheet-like extensions [37]. However, the main issue in the identification of CFs is the lack of a cell specific marker. Commonly used markers for identification of CFs include the discoidin domain receptor 2 (DDR2), periostin (POSTN), vimentin, platelet derived growth factor receptor (PDGFR), CD90/Thy-1 and fibroblast-specific protein 1 (FSP1) [38]. However, none of these proteins are uniquely or continuously expressed in CFs. For example, DDR2 is also expressed in leukocytes [39], POSTN expression is low in non-diseased hearts, and FSP1 expression in mouse models of cardiac fibrosis is not primarily seen in CFs, but rather haematopoietic and vascular cells [40]. The identification of a definitive CF cell-specific marker is crucial for future fibroblast research.

The main role of CFs is to maintain and regulate the ECM, which is important for structural support, signal transduction, and fluid movement [41]. CFs are also involved in fibrotic scar maturation during wound healing post-myocardial injury. Following acute myocardial infarction (MI), CFs become activated, proliferate rapidly, migrate to the site of injury, and express contractile proteins such as α-smooth muscle actin (α-SMA) [42]. These activated CFs are called myofibroblasts and have an increased ability to produce and deposit ECM components, leading to fibrotic accumulation [43]. ECM secretion by myofibroblasts initially causes adaptive fibrosis, which is crucial to protect the structural integrity of the heart, before the fibrotic scar later matures due to the stiffening of secreted collagen at the site of injury [44]. CFs also synthesize and secrete bioactive molecules that are involved in paracrine/autocrine signalling during fibrosis [45]. Pro-inflammatory cytokines interleukin (IL)-6, IL-1β and tumour necrosis factor-α (TNF-α) have diverse effects on CFs. TNF-α increases CF proliferation, migration and pro-inflammatory cytokine expression, but decreases collagen synthesis; IL-1β stimulates ECM degradation and CF migration, but reduces CF proliferation; while IL-6 reduces CF collagen synthesis [45]. The pro-fibrotic cytokine transforming growth factor-β (TGF-β) increases fibrillar collagen, fibronectin and proteoglycan synthesis, and has been reported as a specific stimulus needed for CF differentiation into myofibroblasts. Angiotensin II (Ang II) can also induce CF differentiation [45], whilst the vasoactive peptide endothelin-1 (ET-1) is also a pro-fibrotic mediator that enhances CF proliferation and ECM deposition [46]. Vascular endothelial growth factor (VEGF), secreted by CFs in response to hypoxic or inflammatory stimuli, acts on endothelial cells to induce angiogenesis [45].

### 2.2. Fibroblast Connexins and Cell-Cell Coupling

CFs express several connexin isoforms, with distinct expression patterns in different cardiac regions, disease conditions, and stage of development. Normal adult mouse ventricular CFs express Cx40 and Cx43 in culture [47], while neonatal rat CF cultures express Cx43 and Cx45 [48]. Cx43 has also been observed in adult rabbit ventricular CFs in situ [49]. CFs have been shown to express Cx40 and Cx45 in rabbit SAN tissue [50], Cx40 and Cx43 in the rabbit atrium [49], and Cx40 in the rabbit AVN [51]. Fibroblast connexin expression has also been investigated following cardiac injury, with Cx43 and Cx45 identified in sheep infarct ventricular tissue [52] and Cx43 detected in CFs isolated from post-MI rat hearts [53]. Importantly, CF connexin expression increases following cardiac injury [51,53], indicating a potential role for CF connexins in myocardial remodelling.

Several studies have shown that CFs establish functional gap junctional channels with adjacent CFs as well as with neighbouring CMs, both in vitro and in situ. Functional coupling between ventricular CFs in culture occurs through Cx40, Cx43, or Cx40/43 heterotypic gap junctions [47]; while CF-CF coupling in the SAN is mediated by Cx40 in fibroblast-rich areas devoid of CMs and by Cx45 in regions of the node where fibroblasts intermingle with CMs [50]. Growing evidence indicates that CFs directly communicate with CMs through connexins, with Cx43 [53] and Cx45 [48] identified as the isoforms involved in coupling between neonatal CMs and CFs in co-cultures, and Cx45 supporting CF-CM coupling in SAN tissue [50]. In situ, functionality of this coupling was confirmed by dye transfer assays using the gap-junction-permeable dye Lucifer yellow [50], while in vitro was confirmed by fluorescence recovery after photobleaching of calcein-AM [53], and the observation of successful conduction of electrical impulses between groups of CMs interconnected by fibroblast inserts up to 300 μm in length [48]. Despite the above evidence, whether CF-CM coupling exists in vivo remains subject of debate. A recent study by Quinn et al. [54] provides the first direct proof of functional coupling between non-myocytes and CMs in the whole heart. Using optogenetic tools, they monitored the electrical activity of non-myocytes in Langendorff-perfused mouse hearts and found that non-myocytes displayed CM-like action potentials at the border zone of cryo-injured hearts, indicating the presence of electrotonic coupling between non-myocytes and CMs in native myocardium [54]. This study also supports previous indirect evidence of CF-CM coupling in post-infarct rabbit hearts, where cardiac electrical impulses propagated into scar tissue despite the chemical ablation of surviving endocardial CMs [55]. Further, the presence of connexins in CFs within the infarct suggested CFs may be involved in impulse conduction through gap junction-mediated electrical coupling with CMs [52,56]. These studies highlight the possibility for CF-CM coupling in the ventricles, specifically post-MI to electrically link infarct and non-infarct regions of the heart.

### 2.3. Role of Fibroblasts in Cardiac Electrophysiology

In addition to their biochemical and structural functions, CFs participate in mechano-electrical feedback and can directly contribute to cardiac electrophysiology [49]. CFs can affect electrophysiology passively, for example by acting as obstacles to the spread of electrical activity. Following myocardial injury, CF proliferation and collagen accumulation leads to densely fibrotic ventricular scars that both mechanically and electrically separate surviving bundles of CMs [57]. This can cause arrhythmia through the dense fibrosis first creating areas of conduction block, whilst second, fibrosis can force electrical impulses to take a convoluted path through the separated CM bundles, and even reduce conductivity between CMs, slowing conduction [57]. Further, the development of new treatments targeting fibrosis, reducing collagen deposition and myofibroblast proliferation, show promise for reducing cardiac fibrosis-based arrhythmia [58]. However, the discovery that CFs express connexins and form functional gap junctions with CMs supports the hypothesis that CFs may play a more active role in modulating cardiac electrophysiology. CFs can affect CM electrophysiology through both acting as long-range conductors and actively affecting the action potential properties of CMs [59]. The high membrane resistance and low capacitance of CFs make them suitable to act as conductors for long-range signal transmission [60], whilst CFs can modulate CM electrophysiology due to having a more positive resting membrane potential [59].

Active CF modulation of CM electrophysiology through CF-CM gap junctional coupling has mainly been demonstrated in vitro and in silico. Miragoli et al. [61] demonstrated that coating strands of neonatal rat CMs with myofibroblasts caused slowed conduction as a result of CM depolarisation, Na^+^ channel inactivation, and a slower inward upstroke current [61]. Further, a follow-up study showed co-culturing myofibroblasts and CMs led to ectopic CM automaticity [62], whilst inhibition of myofibroblast-CM coupling in vitro through myofibroblast Cx43 knockdown was found to be anti-arrhythmic [63]. Cx43 knockdown reduced CM-myofibroblast coupling, and prevented the increase in CM maximum diastolic potential, CM automaticity, changes to conduction velocity and prolonged repolarisation, reducing the incidence of re-entrant tachycardia as a result [63]. This is particularly important as myofibroblasts isolated from infarct adult rat hearts have been shown to exhibit higher Cx43 expression and functional coupling with CMs than normal fibroblasts in culture, suggesting they have greater arrhythmogenic potential [53]. A study combining myofibroblast-CM co-cultures and computer simulations looked at the effect of myofibroblast density and level of CM-myofibroblast coupling on conduction velocity. They found that increasing the density of myofibroblasts, or increasing the level of CM-myofibroblast coupling, increased conduction velocity [64]. Together, these results suggested that at low levels of coupling myofibroblasts act as charge sinks to slow conduction velocity, but at higher levels of coupling myofibroblasts instead act as conductors to facilitate conduction to downstream CMs [64].

There has also been indirect evidence of CFs acting as conductors in vivo. Mahoney et al. [65] identified Cx43 gap junctions between CMs and non-myocytes as a possible route for electrical signal transmission in the ventricles, after optically mapped electrical signals were observed in both the ventricular scar region and surrounding healthy myocardium of wild-type mice following cardiac injury [65]. However, electrical signals in the ventricular scar of fibroblast-specific protein-1 driven conditional Cx43 knockout (Cx43FSP1KO) mice (a model where Cx43 is only knocked out in FSP1 positive cells to reduce Cx43 expression in non-myocytes but not CMs) were significantly reduced compared to wild-type mice, suggesting that deletion of Cx43 from FSP1 positive cells reduced the coupling between the scar and uninjured myocardium [65]. Direct current delivery to the scar of wild-type mice was shown to electrically excite the uninjured myocardium, whilst electrical impulses could also conduct across scars [65]. Although these findings support an active role of non-myocytes in cardiac electrophysiology, interpretation of the results is difficult due to the lack of specificity of FSP1 expression. FSP1 can be expressed in CFs, haematopoietic and vascular cells [40], and multiple cell types, aside from CFs and myofibroblasts were present in the injured myocardium of the mice [65]. Therefore, whilst demonstrating that CMs and non-myocytes in ventricular scars are electrically coupled in a Cx43-dependent manner, identification of cell specific markers is needed to provide more information on which non-myocytes couple to CMs.

Whereas gap junctional coupling of ventricular CFs and CMs has been extensively investigated in vitro and recently confirmed in vivo, coupling between atrial CFs and CMs is less understood. Atrial fibrillation (AF) is the most common sustained form of clinical arrhythmia, with atrial fibrosis a clinical hallmark of AF and myofibroblast content increasing more in the atria than the ventricles following congestive heart failure [66]. Computer models have been used to show the potential effect of coupling between atrial CFs and CMs. At the single-cell level, atrial CF coupling with CMs increases the resting membrane potential of CMs, decreases upstroke velocity, and prolongs the action potential duration. Further, when more than 10 fibroblasts are coupled to a CM, the CM is unable to attain the activation threshold for the upstroke Na^+^ current and fails to generate an action potential [67]. A 2D-model of CF-CM coupling has shown CF coupling could disturb the wavefront of atrial excitation and lead to the formation of re-entrant arrhythmias [67]. Targeting of atrial CF-CM coupling for catheter ablation has also been shown to terminate spiral-wave re-entry earlier in AF [68]. Electromechanical coupling of atrial CMs with CFs and a stretch-activated ion channel current has also been shown to modulate atrial CM excitability and action potential morphology [69], whilst heart failure (HF), a clinical syndrome where ventricular filling and blood ejection is impaired due to structural and functional defects in the heart [70], has been shown to cause K^+^ ion channel remodelling in CFs and alter spiral-wave dynamics when CFs are coupled to CMs [71]. Therefore, coupling between CFs and CMs in the atria could contribute to AF, but requires further research in biologically relevant samples.

Altogether, these studies show the potential of CFs/myofibroblasts to contribute to electrical activity in the heart through direct gap junctional coupling with CMs, acting as both conductors and modulators of cardiac electrophysiology. This could be particularly important for treatment of post-MI ventricular arrhythmia, the most common cause of sudden cardiac death post-infarction [72], as CMs and myofibroblasts come in to close contact at areas such as the infarct border zone [53], and in AF which is the most common form of clinical arrhythmia.

### 2.4. Fibroblasts, Connexins and Myocardial Remodelling

CFs play important roles in the wound healing and remodelling response to inflammation in the heart. Several studies have shown Cx43 contributes to CF differentiation, proliferation and migration post-MI. CFs can differentiate in to α-SMA positive myofibroblasts during cell culture, or be induced to differentiate by TGF-β [73], a central mediator of the inflammatory and fibrotic response post-infarction [74]. TGF-β treatment of cultured CFs increases their expression of α-SMA and Cx43, whilst knockdown or overexpression of Cx43 decreases or increases the expression of α-SMA in CFs respectively [73]. Therefore, these results suggest that Cx43 plays a role in the phenotypic conversion of CFs to myofibroblasts in response to TGF-β signalling. This has also been seen in vivo in left anterior coronary artery ligation MI model mice. Cx43-deficient mice show reduced differentiation of CFs to myofibroblasts compared to wild-type mice following injury, as well as delayed scar formation and fibrotic remodelling [75]. Interestingly, Cx43-deficient mice also displayed reduced fibrosis and adverse dilatation 4-weeks post-MI, and were said to have received beneficial post-MI remodelling. These beneficial effects of Cx43 knockout on post-MI remodelling were also attributed to decreased TGF-β signalling [75].

CF proliferation and migration are important parts of the wound-healing process post-MI. An inverse relationship between Cx43 expression and CF proliferation has been shown in vitro, with reduced Cx43 expression increasing CF proliferation, and increased Cx43 expression reducing CF proliferation [76]. Connexin involvement in fibroblast migration and wound healing has been well established outside the heart [77,78,79,80], although little evidence exists for connexin involvement in CF migration. One study has shown Cx43 can affect CF migration in vitro. Treatment of CFs surrounding a spheroid of CMs with α-connexin carboxyl-terminus (αCT1), a mimetic peptide of the carboxyl-terminus (CT) region of Cx43, caused the CFs to migrate to the centre of the spheroid and displace CMs [81]. The αCT1 peptide interferes with the interaction between the Cx43 CT and zonula occludens-1 (ZO-1), an anchoring protein that controls the cellular distribution of Cx43 [82]. This Cx43 CT and ZO-1 interaction also facilitates hemichannel function, as disassociation of this interaction increases the contribution of Cx43 hemichannels to Cx43 gap junctions [83]. Therefore, increasing gap junctional communication between CFs or inhibiting Cx43 hemichannels may induce CF migration during wound healing post-MI.

Fibroblast connexin hemichannels have also been implicated in contributing to myocardial remodelling following cardiac injury. Blockade of Cx43 hemichannels with the peptide Gap26 has been shown to be protective during ischemia-reperfusion injury [84], with further research specifically implicating myofibroblast Cx43 hemichannels [85]. Neonatal rat heart myofibroblasts cultured in ischemia-like conditions were shown to display reduced gap junction communication but increased hemichannel opening and cell death, with Gap26 preventing this hemichannel opening and reducing infarct size [85]. Finally, connexin hemichannels also facilitate adenosine triphosphate (ATP) release from CFs. ATP is released in the heart in response to myocardial injury in order to promote phagocyte recruitment and initiate pro-fibrotic responses [86]. Physical perturbation of CFs by culturing them in hypotonic medium leads to increased ATP release, although this can be prevented by increasing extracellular Ca^2+^, which blocks hemichannel permeability, or by knockdown of Cx43 and Cx45 [86]. This suggests that ATP released from CFs via Cx43 or Cx45 hemichannels can contribute to pro-fibrotic responses following myocardial injury, such as CF differentiation to myofibroblasts and increased collagen synthesis, through activation of P2Y_2_ receptors and stimulating ERK signalling [86].

**Figure 1:**
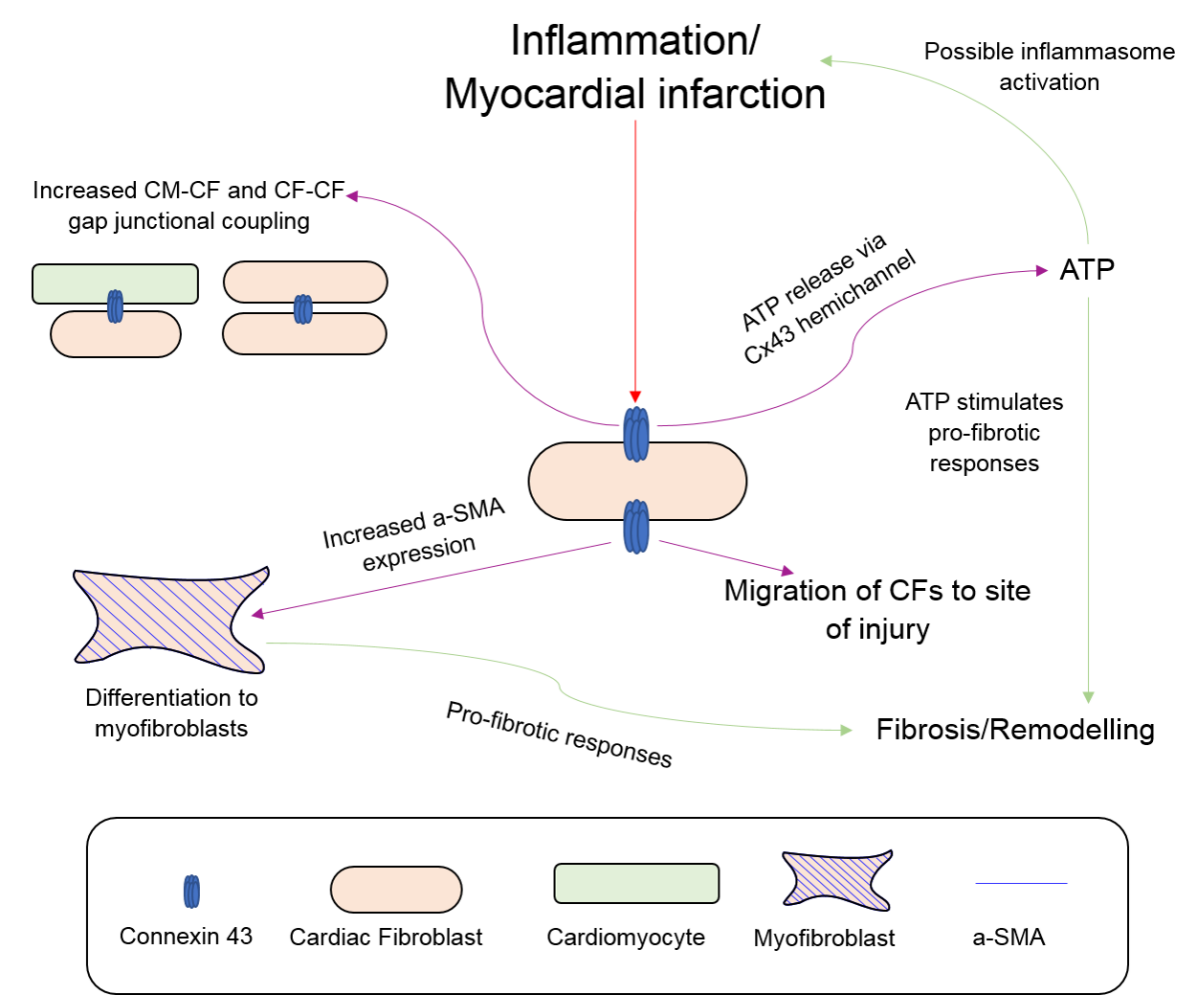
Connexin 43-mediated signalling in cardiac fibroblasts following inflammation/myocardial infarction. Red arrow = Inflammatory stimulus. Purple arrow = Connexin 43-mediated primary response to stimulus. Green arrow = Secondary responses following connexin 43-mediated signalling. Abbreviations: Cx = Connexin. CM = Cardiomyocyte. CF = Cardiac Fibroblast. ATP = Adenosine Triphosphate. α-SMA = α-Smooth muscle actin.

Another potentially important role of CF connexin hemichannels is the regulation of the inflammasome pathway. HF is associated with low-grade chronic inflammation that is mediated by danger-associated molecular patterns (DAMPs), molecules indicating cell damage, and the nucleotide oligomerisation domain (NOD)-like receptor protein-3 (NLRP3) inflammasome pathway [87]. The NLRP3 inflammasome is a complex of proteins that recognise DAMPs and trigger or amplify the inflammatory response through pro-inflammatory cytokine maturation [87]. Activation and amplification of the NLRP3 inflammasome in retinal pigment ECs has been shown to be mediated by an autocrine loop, with Cx43 hemichannel-mediated ATP release inducing assembly of the NLRP3 and cytokine release [88]. This Cx43 hemichannel-mediated autocrine loop has not been shown in CFs, but substantial evidence exists to suggest a similar mechanism may happen following cardiac injury. NLRP3 is expressed in CFs and has been shown to facilitate the differentiation of CFs to myofibroblasts and increase fibrosis in vivo [89]. Moreover, NLRP3 is up-regulated in CFs following ischemia-reperfusion injury and contributes to infarct size and fibrosis [90,91], whilst inflammatory activation of CFs with lipopolysaccharide (LPS) also increases extracellular ATP and facilitates NLRP3 inflammasome formation [90]. Therefore, given the role the NLRP3 inflammasome plays in CF differentiation and remodelling following ischemia-reperfusion, and how CF Cx43/Cx45 hemichannels facilitate ATP release [86] and CF Cx43 hemichannel blockade following ischemia-reperfusion is protective [85], it is likely that CF connexin hemichannels play a role in the initiation and amplification of inflammation during HF and MI. This is an area for future research and could better clarify the inflammatory mechanisms during HF and MI. Figure 1 summarises the potential involvement of Cx43 in the response of CFs to inflammation/myocardial infarction.

In summary, connexins appear to modulate the function of CFs during post-MI wound healing. CFs isolated from the infarct heart have previously been shown to display upregulated Cx43 expression and increased intracellular communication [92], with this increased Cx43 possibly leading to myofibroblast differentiation, CF migration, and adverse remodelling seen post-MI. However, increased Cx43 expression was shown to reduce CF proliferation, the opposite of what happens post-MI, suggesting further mechanisms need to be elucidated. There is also potential for ATP released via Cx43 hemichannels to initiate and amplify the inflammatory response during HF or post-MI, and further stimulate pro-fibrotic responses.

## 3. Connexins and Endothelial Cells

### 3.1. Roles of Endothelial Cells in Cardiac Muscle and the Vasculature

Endothelial cells form the cardiac endothelium, the lining in the chambers of the heart and the coronary vasculature [9]. The cardiac endothelium is involved in signal transduction of neurotransmitters, mechanical stimuli and hormones, and has been reported to modulate the contractile function of CMs through paracrine signalling of nitric oxide (NO) and ET-1, among others [9]. NO in ECs is produced by endothelial NO synthase (eNOS), with NO affecting the onset of myocardial relaxation [93]. ET-1 has the opposite effect, binding to ET_A_ receptors on CMs causing CM constriction [93]. In the normal myocardium, the dense capillary network guarantees close proximity between ECs and CMs, facilitating interactions between the two cell types [93]. The cardiac endothelium also has roles in control of heart size and angiogenesis, with suggestions that these two processes may be linked [94].

Endothelial cells also form a lining along every blood vessel in the human body, the vascular endothelium. The vascular endothelium is involved in regulation of vascular contraction, through similar NO and ET-1 paracrine signalling to that seen in the cardiac endothelium [95]. The vascular endothelium also plays roles in regulating thrombosis and thrombolysis, as well as platelet/leukocyte interaction with the vessel wall [96]. Under normal conditions, ECs work to prevent thrombosis by means of anti-coagulant and anti-platelet mechanisms, but upon injury this is changed to help restore vascular integrity. Induction of pro-coagulant factors is important in the formation of fibrin, whilst platelet and leukocyte adhesion are important for recruitment of inflammatory cells to sites of injury [96].

### 3.2. Endothelial Cell-Cardiomyocyte Gap Junctional Signalling

ECs express three types of connexion, Cx40, Cx43, and Cx37 [97], with the first evidence for EC-CM gap junctions provided by Narmoneva et al. [98]. This study found ECs contributed to both the spatial organisation and survival of CMs, as well as synchronising CM contraction as cultures containing both CMs, and ECs showed a larger area of synchronised contraction than cultures of CMs only [98]. ECs were also shown to increase the expression of Cx43 in CMs in a VEGF-dependent manner, and increase both CM-CM and EC-CM coupling [98]. EC-CM uncoupling has been suggested to contribute to cardiac arrhythmia and failure in patients with hyperhomocysteinemia (HHcy). HHcy leads to elevated levels of homocysteine (Hcy), and has been associated with altered myocardial conduction and sudden cardiac death [99]. Elevated levels of Hcy increased Cx43 degradation and peri-capillary fibrosis in mice, leading to disconnection between ECs and CMs. The elevated Hcy also caused arrhythmogenesis, and impaired ET-1 and NO-mediated regulation of cardiac contraction. These effects were ameliorated with MK-801 treatment, a NMDA-R1 antagonist, suggesting a role for the NMDA-R1 in EC-CM uncoupling and arrhythmogenesis [99]. However, as both Cx43 degradation and fibrosis contributed to the EC-CM uncoupling, it is unknown what the contribution of Cx43-mediated uncoupling is towards arrhythmogenesis in HHcy, and should be investigated further.

Endothelial Cx40 has been shown to be beneficial after ischemia-reperfusion injury. The harmful effects of Cx43 hemichannels in CFs during ischemia-reperfusion have been explored earlier, but interestingly Cx40 in ECs is believed to confer cardioprotective effects. EC-specific deletion of Cx40 in mice causes neutrophil infiltration, increased infarct size, and increased cell death following ischemia-reperfusion injury, although no deleterious effects have been associated with Cx37 deletion [100]. The Cx40-mediated protective effect is thought to be mediated by CD73 signalling, as methotrexate activation of CD73 in Cx40-knockout mice reduced neutrophil infiltration and infarct size [100]. This effect is believed to arise from anti-adhesion signalling by CD73, which propagates through Cx40 gap junctions between ECs to reduce leukocyte adhesion [101]. Similar effects of Cx40 deletion on recovery have been seen after post-ischemic hindlimb injury, where deletion of Cx40 reduced limb recovery and survival after ischemia [102] as a result of reduced tissue perfusion, arteriogenesis, and increased inflammatory response [103].

### 3.3. Endothelial Cells, Connexins, and Vasomotor Control

Gap junctions between ECs are important for allowing communication between cells to coordinate EC function in the vasculature. For example, knockdown of Cx37, Cx40, or Cx43 leads to reduced intercellular communication between human umbilical vein endothelial cells (HUVECs), impairing angiogenesis in matrigels as a result [104]. Gap junctional coupling between ECs has been shown to play a central role in coordinating the Ca^2+^ response of the endothelium to vasoactive agonists, with histamine stimulation of HUVECs causing an initial rapid increase in intracellular Ca^2+^ that slowly spreads to neighbouring cells, a spread that is prevented by gap junctional blockade. The same effects have also been observed in mouse aorta after ATP stimulation [105]. Endothelium Ca^2+^ increase plays an important role in NO production by eNOS, with gap junctional blockade in HUVECs also shown to reduce NO production in cells after histamine treatment [106]. Further to this, knockout of EC Cx43 in mice has caused hypotension and elevated NO plasma levels, suggesting Cx43 also regulates NO production [97]. Direct interaction between EC Cx37/40 and eNOS has been suggested, with Cx37 and eNOS found to colocalise at points of cell-cell contact in mouse and human EC lines, and Cx37 antisense treatment increasing NO production [107]. Co-localisation of Cx37, Cx40, and eNOS at points of cell-cell contact has also been reported in the aortic endothelium of Cx40^+/+^ mice. Deletion of Cx40 reduced NO production, ATP-induced endothelium relaxation, and eNOS expression [107]. However, whilst only Cx40 was knocked out, a concurrent reduction in Cx37 was also observed, suggesting alterations in both Cx40 and Cx37 contributed to the reduced eNOS expression [107]. Interestingly, the above studies show opposing effects to NO production when Cx37 or Cx40 is reduced. However, given the different origin of ECs used, with venous ECs investigated in the first study and arterial ECs in the second study, this suggests that connexins could have different roles in the vasomotor control of veins and arteries and is a possible area of future research.

ECs have been reported to affect SMCs relaxation via direct gap junctional coupling. Electron microscopy images have revealed areas of close contact between vascular endothelium and smooth muscle that contain myoendothelial gap junctions [108]. The role of these heterocellular gap junctions in the electrical and chemical coupling between ECs and SMCs has been demonstrated in the vascular wall of arteries. Simultaneous microelectrode recordings of ECs and SMCs in the vessel wall revealed both cells had identical resting membrane potentials, and in response to current injection showed simultaneous, equivalent changes in membrane potential [109]. Importantly, bidirectional electrical coupling was observed, with depolarising and hyperpolarising currents injected in one cell leading to depolarisation or hyperpolarisation of the other [109]. Li et al. [110] however reported unidirectional coupling, with signals flowing from ECs to SMCs in spiral modiolar arteries isolated from the guinea pig cochlea [110]. Therefore, whilst electrical coupling between ECs and SMCs appears to be present and able to transmit signals to regulate vasomotor control, the co-ordination of this coupling is not well understood.

### 3.4. Endothelial Cells, Connexins and Atherosclerosis

Atherosclerosis is a disease which can eventually lead to thrombosis and possible stroke, MI, or sudden cardiac death [111]. Atherosclerosis begins with subendothelial accumulation of lipoproteins which triggers an inflammatory response, and subsequent activation of cells in the vascular wall and recruitment of monocytes and macrophages, forming atherosclerotic plaques (atheromas) [112]. Most of these atherosclerotic plaques do not cause vascular disease, but some vulnerable plaques have necrotic cores which can lead to plaque rupture and promotion of vessel thrombosis [112].

**Figure 2:**
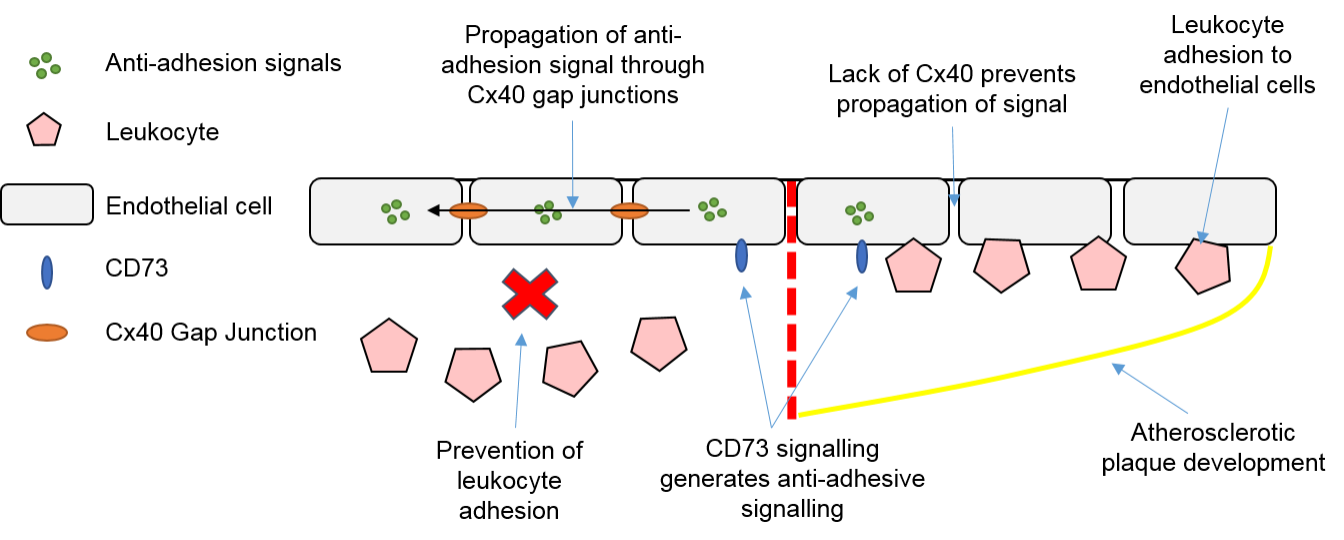
Connexin 40 gap junctions between endothelial cells are atheroprotective through preventing leukocyte adhesion. Yellow line = atherosclerotic plaque border. Abbreviations: Cx = Connexin.

Evidence for multiple EC connexin-based involvements in atherosclerosis has been suggested. This includes anti-inflammatory and anti-adhesion signalling mediated by Cx40 gap junctions, as mentioned earlier. Knockout of Cx40 in mice causes increased atherosclerosis, inflammation, and vascular cell adhesion molecule-1 (VCAM-1) expression; VCAM-1 is involved in recruitment of monocytes during atherosclerosis [101]. The protective role of Cx40 is believed to come from its ability to propagate and synchronise anti-adhesion/inflammatory signals generated by CD73 across ECs, therefore inhibiting leukocyte recruitment and reducing atherosclerosis [101]. This atheroprotective role of Cx40 is illustrated in Figure 2. There is also a potential atheroprotective role of Cx37, which is highly expressed in ECs in the healthy artery but is lowly expressed in ECs overlying atheromas [113]. High laminar shear stress (HLSS) in vessels increases the expression of Cx37 in ECs and coupling between ECs. This coupling is atheroprotective by helping to synchronise ECs and maintain them in an inactive state, therefore preventing endothelial dysfunction [113]. However, the expression of the Cx40 and Cx37 in ECs can be reduced by hyperlipidaemia, a condition that accelerates the onset of atherosclerosis [114]. Treatment with simvastatin, a lipid-lowering drug reported to decrease atherosclerosis progression, has been shown to aid the recovery of Cx37 expression, but not of Cx40 [114]. This indicates a possible link between the expression of atheroprotective connexins and lipid levels in the progression of atherosclerosis. More research in to the mechanisms behind how lipid levels modulate connexin expression could provide new insights into the initiation of atherosclerosis, and the role connexins play in this.

Furthermore, Cx37 has also been shown to have anti-proliferative effects and may be protective against neointimal hyperplasia. Neointimal hyperplasia is the thickening of the intimal layer of the artery, resulting in lesions composed mainly of SMCs and proteoglycans. These hyperplastic lesions can be pre-cursor sites for the development of atherosclerosis [115], and begin to develop after endothelial dysfunction causes vascular SMCs to de-differentiate and proliferate [116]. Shear-stress induced endothelial quiescence and reduced proliferation has been shown in HUVECs as a result of Notch-Cx37-p27 signalling, promoting cell cycle arrest and expression of arterial genes to allow arterial specification [117]. Additionally, the overexpression of Cx37 in vascular SMCs has also been shown to inhibit proliferation, whilst vascular SMCs isolated from Cx37-deficient (Cx37^−/−^) mice proliferate faster than those from wild-type mice [116]. Knockout of Cx37 was also shown to exacerbate, but delay the onset of, neointimal hyperplasia, whilst the development of neointimal hyperplasia correlated with increased cell proliferation in Cx37^−/−^ mice [116]. This anti-proliferative effect of Cx37 was first shown by Burt et al. [118], where induced expression of Cx37 delayed cell cycle progression and reduced proliferation in rat insulinoma cells [118]. A possible mechanism explaining this anti-proliferative effect is through differential phosphorylation of Cx37 CT regulating gap junctional conductance, with reduced gap junctional conductance being associated with both reduced proliferation and apoptosis in rat insulinoma cells [119].

There has also been evidence that Cx32 can provide protection against vascular inflammation. Overexpression of Cx32 in HUVECs reduces IL-6 and monocyte chemotactic protein-1 (MCP-1) secretion following TNF-α treatment, and knockout of Cx32 in mice leads to increased serum concentrations of inflammatory cytokines following LPS injection [120]. These results suggest Cx32 can regulate inflammatory cytokine production both in vitro and in vivo, and therefore targeting Cx32 could provide a novel treatment route to prevent atherosclerosis and other inflammatory vascular disorders [120]. Finally, Cx32 and Cx43 have both been implicated in arterial stiffening, a cholesterol-independent risk factor for cardiovascular events following atherosclerosis [121]. This stiffening is thought to arise from gap junctional blockade increasing focal adhesion formation following TNF-α treatment. Integrin-dependent focal adhesion formation leads to cell stiffening due to the re-arrangement and contraction of actin [121].

Altogether, these studies support the idea that connexins and gap junctions play a role in atherosclerosis and the associated endothelial dysfunction, and could be suitable therapeutic targets in the treatment of atherosclerosis and other vascular diseases.

## 4. Connexins and Macrophages

### 4.1. Roles of Macrophages in the Heart and Vasculature

Macrophages are one of the main cells involved in the innate immune response, with both blood monocyte-derived macrophages and tissue-resident macrophages existing [122]. Tissue-resident macrophages are present in tissues from embryogenesis, whilst blood monocyte-derived macrophages respond to injury and infection, infiltrating to sites of injury [123]. Tissue-resident macrophages can be found in both the heart and larger arteries, and under normal physiological conditions are involved in phagocytosis of bacteria and debris. Cardiac macrophages are also involved in neonatal cardiac regeneration following injury due to their ability to promote angiogenesis [123]. However, the role of macrophages following injury is much greater, specifically after MI and in the development of atherosclerosis. Following MI and cell death, inflammatory signals recruit macrophages and monocytes to the site of injury, where they contribute to early and late stage MI healing. Early-stage healing involves clearing damaged tissue and secreting proteolytic enzymes, whilst late-stage healing involves promoting myofibroblast accumulation, collagen deposition, and angiogenesis [124]. Infiltrating monocytes may also cause fibronectin release from the ECM, fibronectin being important in scar stabilisation [124]. However, whilst aiding wound healing post-MI, macrophages contribute to the progression and worsening of atherosclerosis. Macrophages contribute to every stage of atherosclerosis, from ingestion of deposited lipoproteins to form foam cells which persist in plaques, to the secretion and production of pro-inflammatory cytokines and matrix metalloproteinases, which de-stabilise and eventually rupture atherosclerotic plaques [125].

### 4.2. Macrophages, Connexins and Electrical Conduction in the Heart

In addition to the above functions, macrophages have recently been shown to play a role in cardiac conduction in the AVN. The AVN is the only electrical connection between the atria and ventricles and is therefore crucial in cardiac electrical conduction. Hulsmans et al. [126] found that cardiac macrophages were abundant in both mice and human AVNs, exhibiting spindle-shaped appearances and long protrusions which formed connections with stromal cells [126]. Real-time qPCR and immunofluorescence analysis of the AVN macrophages found them to express Cx43 at points of contact between macrophages and CMs. Co-cultures of these AVN macrophages and neonatal mouse CMs also showed Cx43 localised at points of cell interaction, therefore suggesting gap junctional coupling between the two cell types [126]. Patch-clamp analysis revealed 23% of these coupled macrophages displayed rhythmic action-potential like depolarisation in culture, whilst a further 23% displayed irregular depolarisation, and the final 54% no depolarisation. Optogenetic stimulation and depolarisation of macrophages was then found to increase AVN conduction, whilst deletion of Cx43 from AVN macrophages slowed conduction, and complete macrophage ablation induced conduction block. This data suggests that Cx43 coupling of macrophages and CMs only partly influences AVN conduction, and macrophages have Cx43-independent roles influencing conduction [126]. However, AVN connexin expression is different between mice and humans, as mice also express Cx30.2, a connexin that forms ultra-small-conductance channels, whilst humans either do not express or express at very low levels Cx31.9, the human orthologue of Cx30.2 [127]. As a result, these differences in connexin expression could lead to differences in AVN conduction between mice and humans, suggesting macrophages may not be involved in human AVN conduction. Therefore, while this is the first study to show direct involvement of macrophages in cardiac conduction, the exact role of connexin-mediated macrophage-CM coupling in normal and pathological conditions is unknown and represents an exciting area for future research.

Macrophages have also previously been suggested to affect conduction through altering Cx43 expression post-MI. Atherosclerotic mice who undergo MI show slowed conduction and post-MI arrhythmias, as well as macrophage infiltration and reduced Cx43 expression. This macrophage infiltration to infarcted areas was shown to correlate with Cx43 degradation, whilst CMs bordered by macrophages showed clear Cx43 internalisation and degradation [128]. Mice also displayed elevated serum IL-1β, a cytokine secreted by macrophages which has been known to degrade Cx43, suggesting secretion of IL-1β by macrophages was responsible for the observed Cx43 degradation [128]. Therefore, macrophages appear to have direct and indirect roles in modulating cardiac conduction, both of which are associated with Cx43 gap junctions.

### 4.3. Macrophages, Connexins, and Atherosclerosis

**Figure 3:**
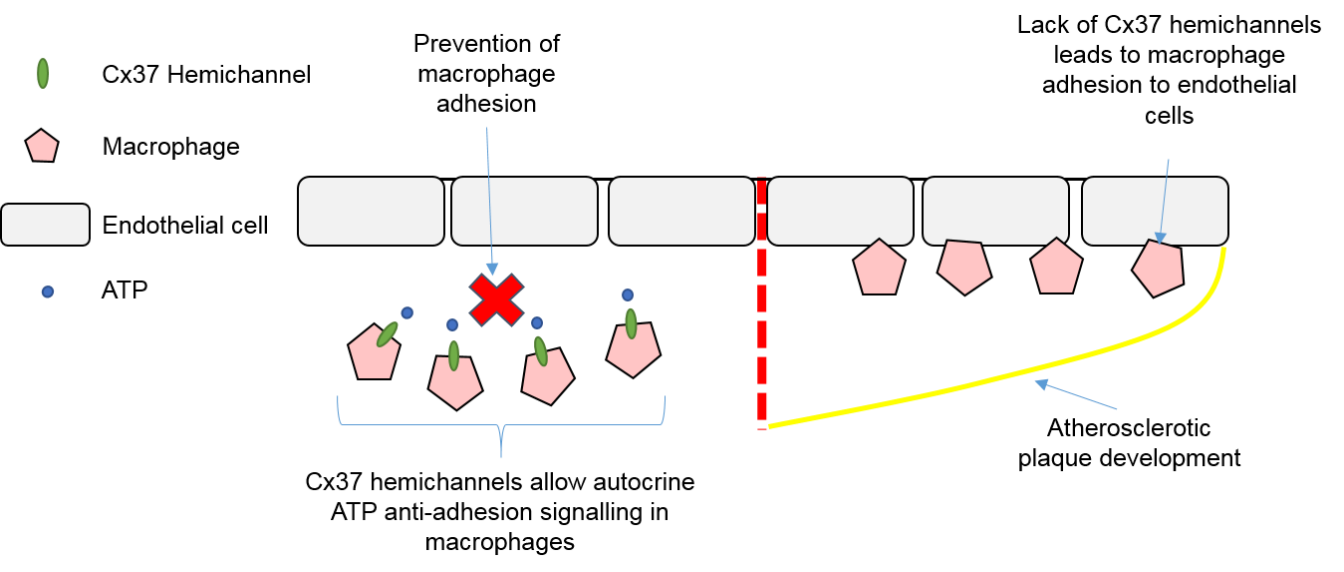
Autocrine ATP signalling via connexin 37 hemichannels prevents macrophage adhesion to endothelial cells. Yellow line = atherosclerotic plaque border. Abbreviations: Cx = Connexin. ATP = Adenosine Triphosphate.

The role of macrophages in atherosclerosis development has been well established, with connexins thought to contribute to leukocyte adhesion and recruitment. Macrophage/monocyte Cx37 hemichannels have been shown to be atheroprotective through regulating leukocyte recruitment and adhesion to the endothelium, with Cx37 deletion in atherosclerotic mice leading to increased endothelial macrophage adhesion and presence of macrophages in atherosclerotic plaques [129]. This adhesive effect of Cx37 deletion was thought to arise from inhibition of autocrine ATP signalling through Cx37 hemichannels, with degradation of extracellular ATP shown to increase adhesion of Cx37-expressing macrophages to an activated endothelial monolayer, whereas Cx37-deficient macrophages were unaffected [129]. Therefore, it appears Cx37 hemichannels on macrophages can regulate macrophage/monocyte adhesion and the initiation of atherosclerosis through autocrine ATP signalling [129], with this summarised in Figure 3. Macrophage Cx43 has also been implicated in contributing to atherosclerotic plaque development through chemo-attraction of neutrophils, another type of immune cell which accumulates in atherosclerotic plaques [130]. Foetal haematopoietic liver cells were taken from Cx43^+/+^, Cx43^+/−^, and Cx43^−/−^ mice and reconstituted in low-density lipoprotein (LDL) receptor–knockout atherosclerotic mice. Atherosclerotic mice who received Cx43^+/−^ chimeras showed reduced atherosclerosis and presence of neutrophils compared to both Cx43^+/+^ and Cx43^−/−^ chimeras. This was attributed to altered levels of chemoattractant proteins being present in the supernatant of Cx43^+/−^ macrophages as neutrophils showed reduced transwell migration when assayed towards Cx43^+/−^ macrophage supernatant [130]. Therefore, the expression level of Cx43 appears to influence the production of chemoattractant and chemotactic proteins, and future research should be conducted to determine why and how specific levels of Cx43 lead to this irregular secretion [130].

Finally, a novel role of macrophage Cx43 in atherosclerotic development through favouring macrophage phagocytosis of oxidised-LDLs has been suggested [131]. Cx43 has been shown to regulate phagocytosis in macrophages through Fc receptor-induced Ras homolog gene family, member A (RhoA)-dependent rearrangement of the actin cytoskeleton [132]. Therefore, the suggestion is that other receptors could drive phagocytosis, such as scavenger receptors, and this could lead to favoured uptake of oxidised-LDL [131]. LDL has previously been shown to enhance monocyte/macrophage phagocytosis of *A Streptococcus*, with increasing evidence that infection may also contribute to the development of atherosclerosis [133]. However, this has yet to be definitively established, and if Cx43 directs phagocytic uptake via LDL is unknown.

## 5. Non-Myocyte Gap Junctions and Hemichannels as Novel Therapeutic Targets

This review has so far described the roles of non-myocyte connexins, hemichannels, and homocellular/heterocellular gap junctions in the cardiovascular system during health and disease. From potential influence over cardiac electrophysiology to atherosclerotic development, the possibility of developing therapeutics targeting connexins retains large interest. Substantial recent work has been done to develop peptides that specifically target individual connexins, or target gap junctions and hemichannels independently of each other [134]. Examples of these peptides include rotigaptide and αCT1. Rotigaptide works by promoting gap junctional coupling between cells and was originally developed as an anti-arrhythmic therapy [135,136]. Rotigaptide has been shown to both reduce myocardial infarct size and prevent impairment of endothelium-dependent vasomotor function following ischemia-reperfusion injury [137]. The Cx43 CT mimetic peptide αCT1, which was mentioned earlier to promote CF migration, has been shown to reduce arrhythmia following cardiac injury in mice by increasing the number of gap junctions at the infarct border zone [138]. Further to this, αCT1 treatment of submuscular biomedical implants reduces type 1 collagen deposition and number of myofibroblasts around the implant [139], whilst αCT1 treatment of skin wounds leads to faster wound closure, reduced inflammation, swelling, neutrophil recruitment, and granulomas tissue, as well as improved mechanical properties of the skin following closure [140]. Although this has not been investigated in CFs, if similar results are seen then treatment with the αCT1 peptide could improve post-MI healing through reducing fibrosis and inflammation, whilst improving the mechanical strength of the damaged tissue. Therefore, targeting the Cx43 CT could be a useful therapeutic target to improve recovery following MI by reducing the associated arrhythmia and adverse myocardial remodelling.

There is also potential to repurpose drugs currently used for other clinical purposes to target connexins and connexin-based signalling. Two examples of this include simvastatin and methotrexate. Simvastatin, a lipid-lowering drug, has been shown to improve Cx37 expression during hyperlipidaemia and be atheroprotective [113], and could therefore find additional use as treatment to prevent atherosclerotic plaque build-up, although more research is needed to determine how lipid levels and statin drugs regulate connexin expression. Methotrexate is a widely used drug in the treatment of cancer and inflammatory conditions [141], but has also been shown to reduce infarct size following ischemia-reperfusion in Cx40 knockout mice [100]. Down-regulation of Cx40 has been implicated in atherosclerosis as well [101]. Therefore, this suggests methotrexate could find additional uses aiding recovery after ischemia-reperfusion and as an anti-atherosclerotic therapy through bypassing the need for Cx40 gap junctions to propagate anti-inflammatory signals between ECs.

Another possible anti-atherosclerotic treatment involves rutaecarpine, a major quinazolinocarboline alkaloid that is isolated from the traditional Chinese herbal medicine Evodia. Rutaecarpine has been shown to be protective against atherosclerosis through regulating connexin expression in both ECs and monocytes. Pre-treatment of HUVECs with rutaecarpine prevents oxidised-LDL mediated endothelial damage, the reduction in Cx37 and Cx40 expression, and monocyte adhesion [142], whilst a later follow-up study found rutaecarpine pre-treatment of monocytes recovered Cx37 expression and anti-adhesive hemichannel ATP signalling following oxidised-LDL treatment [143]. Preventing endothelial oxidised-LDL damage and monocyte adhesion would therefore be useful to prevent the initiation of atherosclerotic plaque build-up, halting disease progression at its early stages.

Gene therapy could also hold massive potential for treating connexin-related diseases, especially those where loss of connexin expression is associated with disease initiation/progression. For example, AF has been associated with down regulation of Cx43 and slowed conduction. However, atrial delivery of adenoviral vectors containing Cx43 has been shown to increase atrial Cx43 expression and atrial conduction, consequently preventing the development of persistent AF [144]. Therefore, this highlights the ability of gene therapy to restore cardiac connexin expression in disease and reduce arrhythmogenic risk. Some of the potential therapies targeting non-myocyte connexins are summarised in Table 1.

This review has also highlighted possible roles of connexins in non-myocyte populations that have therapeutic value which has yet to be investigated. An example of this is in the possible role of connexin modulation of NO production. We previously mentioned how Cx37 and Cx40 are thought to interact directly with eNOS, and how reduced Cx37/40 expression reduces NO production [107], and therefore targeting this interaction could provide therapeutic benefit. Patients with hypertension have been shown to have reduced plasma NO levels [148], whilst the expression of Cx37 and Cx40 is also reduced in hypertensive animal models [149]. Therefore, investigations in to how Cx37 and Cx40 modulate NO production could lead to the development of treatments which target and facilitate this interaction, increasing NO production and reducing hypertension as a result. Further to this, glyceryl trinitrate (GTN; nitroglycerin) is a common treatment for patients with angina pectoris, MI or HF. The therapeutic benefit of GTN arises from its ability to generate NO and cause vasodilation, restoring blood flow to ischemic hearts [150]. However, sustained GTN treatment has been associated with increased risk of exacerbating cardiac damage, possibly through inactivating the cardioprotective enzyme aldehyde dehydrogenase 2 [150]. Therefore, targeting Cx37 and Cx40 to increase NO production could also be beneficial in the treatment of angina pectoris, MI, and HF through providing the same vasodilatory effect, but avoiding aldehyde dehydrogenase 2 inactivation.

The recent discovery that macrophages contribute to AVN conduction could also be useful for treating arrhythmias and conduction abnormalities that result from inflammation of the heart. The inflammatory diseases lyme carditis, viral myocarditis, and cardiac sarcoidosis have all been associated with cardiac conduction abnormalities [151,152,153], whilst there has also been a reported case of a patient suffering from complete atrioventricular block after the regression of infectious myocarditis [154]. Macrophages also change phenotype and numbers in response to MI and HF, both of which are associated with ventricular arrhythmia and sudden cardiac death [126]. More research is needed to look at the contribution of macrophages to cardiac conduction during health and disease, if macrophages contribute to conduction block or conduction disorders associated with inflammatory diseases, and if macrophages couple with CMs outside of the AVN. Targeting macrophage-CM coupling could prove to be a useful target when treating inflammation-associated conduction abnormalities.

However, the biggest problem to overcome with connexin-based therapeutics is being able to target individual connexins, gap junctions, or hemichannels, in a specific tissue and cell type, as connexins are widely expressed in different tissues. For example, Cx43 is abundantly expressed in more than 35 tissues [155], and in the cardiovascular system alone is expressed in CMs, CFs, ECs and macrophages. Therefore, targeting cell-specific connexins is important so as to not disrupt connexin functions in other cell types and tissues.

## 6. Conclusions

**Figure 4:**
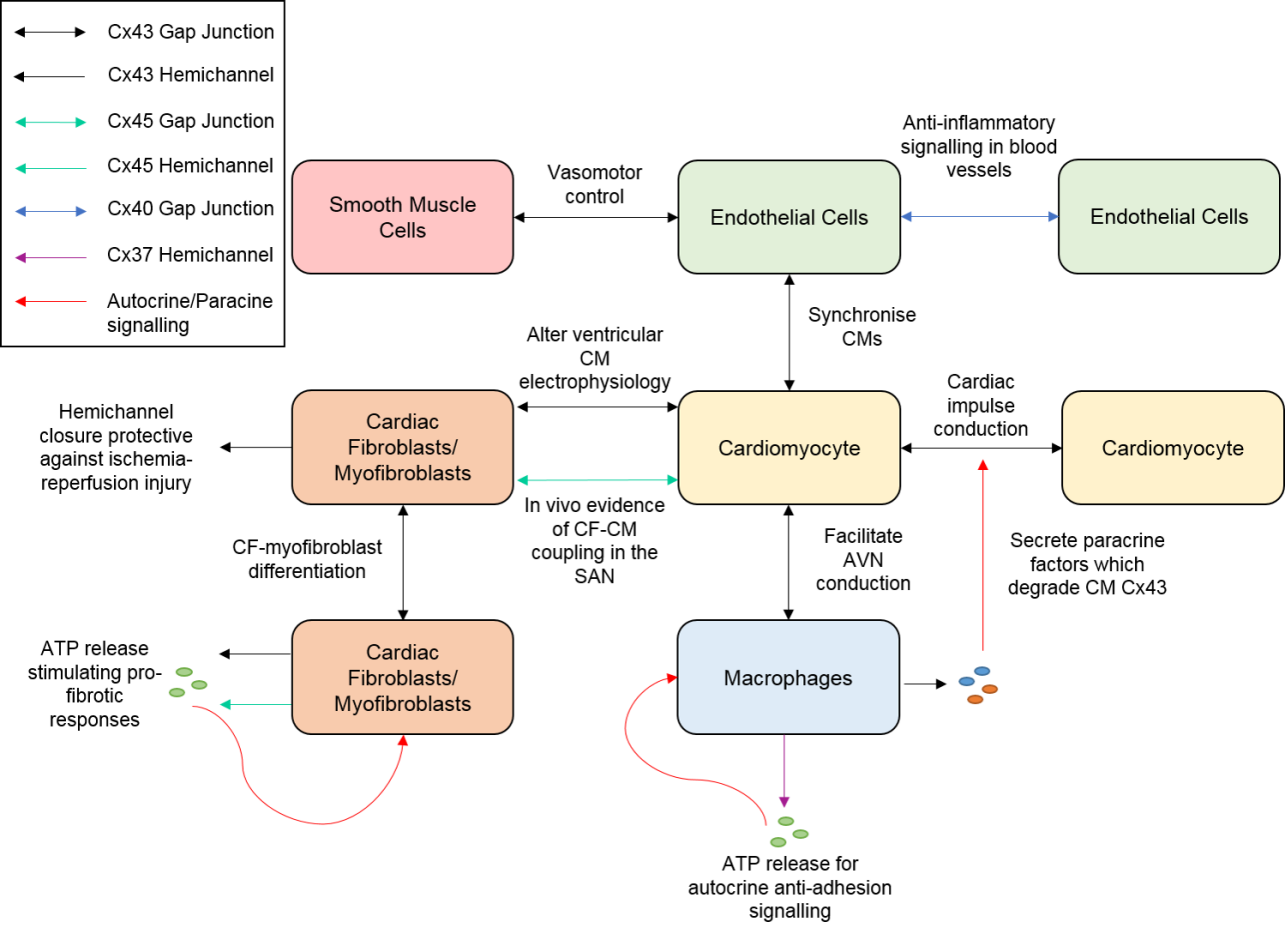
Schematic summary of connexin-based signalling in cardiac non-myocyte populations. Abbreviations: CM = Cardiomyocyte. CF = Cardiac Fibroblast. Cx = Connexin. SAN = Sinoatrial node. AVN = Atrioventricular node. ATP = Adenosine triphosphate.

In conclusion, the evidence for non-myocyte gap junctions and hemichannels contributing to cardiovascular disease grows and is summarised schematically in Figure 4. However, targeting these gap junctions or hemichannels is a promising new research avenue and has potential to develop new therapeutics to reduce arrhythmias, aid recovery from cardiac injury, and prevent the development of inflammatory vascular diseases such as atherosclerosis.

## Figures and Tables

**Table 1:** Potential non-myocyte connexin therapeutics.

Connexin Target	Therapeutic Intervention	Cell Types	Mode of Action	Potential Outcomes
Cx43	Rotigaptide	Cardiomyocytes, endothelial cells	Facilitates Cx43 gap junctional coupling	Anti-arrhythmic [135,136]
				Reduces infarct size and endothelial dysfunction following ischemia-reperfusion [137]
	Gap26	Cardiomyocytes, myofibroblasts	Cx43 gap junction and hemichannel blocker	Reduces infarct size following ischemia-reperfusion [85]
				Improved CM viability following ischemia-reperfusion [84]
	Gap27	Corneal epithelial cells, non-diabetic skin cells	Cx43 gap junction and hemichannel blocker	Improves corneal and skin wound healing [145,146]
	Peptide5	Retinal pigment endothelial cells, potentially cardiac fibroblasts	Cx43 hemichannel blocker	Prevents NLRP3 inflammasome assembly and activation [88]
				Potential to reduce inflammatory response post-MI/HF
	Gap19	Cardiomyocytes	Cx43 hemichannel blocker	Improves CM viability following ischemia-reperfusion [35]
	αCT1	Cardiomyocytes, cardiac fibroblasts	Prevents Cx43 CT and ZO-1 interaction	Induces CF migration in vitro [81]
				Reduces arrhythmia following MI [138]
				Possible regenerative healing, shown in cutaneous wounds [140]
	JM2	Endothelial cells	Cx43 hemichannel blocker	Inhibits ATP release and inflammatory response [147]
	Adenoviral-Cx43 gene therapy	Atrial cells	Increases expression of Cx43	Prevents the development of persistent atrial fibrillation [144]
Cx40	Rutaecarpine	Endothelial cells	Prevents reduction in Cx40 expression	Atheroprotective [142]
	Methotrexate (indirect effect)	Endothelial cells	CD73 activator, anti-adhesive	Methotrexate treatment shown to be atheroprotective [101]
Cx37	Rutaecarpine	Monocytes/Macrophages	Prevents reduction in Cx37 expression	Atheroprotective [143]

Abbreviations: Cx = Connexin. CM = Cardiomyocyte. NLRP3 = NOD-like receptor protein 3. MI = Myocardial Infarction. HF = Heart Failure. CF = Cardiac Fibroblast. CT1 = -connexin carboxyl-terminal. CT = Carboxyl-Terminus. ZO-1 = Zonula Occludens-1. JM2 = Juxtamembrane 2. ATP = Adenosine Triphosphate.

## Abbreviations

CMs	Cardiomyocytes
CFs	Cardiac Fibroblasts
ECs	Endothelial Cells
SMCs	Smooth Muscle Cells
ECM	Extracellular Matrix
Cx	Connexin
SAN	Sinoatrial Node
AVN	Atrioventricular Node
Na^+^	Sodium
K^+^	Potassium
ATP	Adenosine triphosphate
Ca^2+^	Calcium
DDR2	Discoidin Domain Receptor 2
POSTN	Periostin
FSP1	Fibroblast Specific Protein 1
α-SMA	α-Smooth Muscle Actin
MI	Myocardial Infarction
Cx43FSP1KO	Fibroblast-Specific Protein-1 Driven Conditional Cx43 Knockout
TGF-β	Transforming Growth Factor-β
NO	Nitric Oxide
ET-1	Endothelin-1
eNOS	Endothelial Nitric Oxide Synthase
VEGF	Vascular Endothelial Growth Factor
HHcy	Hyperhomocysteinemia
Hcy	Homocysteine
HUVECs	Human Umbilical Vein Endothelial Cells
VCAM-1	Vascular Cell Adhesion Molecule-1
IL	Interleukin
MCP-1	Monocyte Chemotactic Protein-1
TNF-α	Tumour Necrosis Factor-α
LPS	Lipopolysaccharide
RhoA	Ras homolog gene family, member A
HLSS	High laminar shear stress
LDL	Low-Density Lipoprotein
DAMPs	Danger-associated Molecular Patterns
NOD	Nucleotide Oligomerisation Domain
NLRP3	NOD-like Receptor Protein-3
αCT1	α-Connexin Carboxyl-Terminus
ZO-1	Zonula Occludens-1
CT	Carboxyl-Terminus
AF	Atrial Fibrillation
PDGFR	Platelet Derived Growth Factor Receptor
GTN	Glyceryl Trinitrate
JM2	Juxtamembrane 2
